# The Aging Curve: How Age Affects Physical Performance in Elite Football

**DOI:** 10.3390/jfmk10040385

**Published:** 2025-10-03

**Authors:** Luís Branquinho, Elias de França, Adriano Titton, Luís Fernando Leite de Barros, Pedro Campos, Felipe O. Marques, Igor Phillip dos Santos Glória, Erico Chagas Caperuto, Vinicius Barroso Hirota, José E. Teixeira, Pedro Forte, António M. Monteiro, Ricardo Ferraz, Ronaldo Vagner Thomatieli-Santos

**Affiliations:** 1 Biosciences School of Elvas, Polytechnic Institute of Portalegre, 7350-092 Elvas, Portugal; luisbranquinho@ipportalegre.pt; 2 Life Quality Research Center (LQRC-CIEQV), 2001-964 Santarem, Portugal; 3 Research Center in Sports Sciences, Health Sciences and Human Development, 6200-001 Covilhã, Portugal; pedromiguel.forte@iscedouro.pt (P.F.); ricardompferraz@gmail.com (R.F.); 4 Centro de Investigação do Instituto Superior de Ciência Educativas (CI-ISCE), 4560-547 Penafiel, Portugal; 5 Interdisciplinar Graduate Program in Health Sciences, Universidade Federal de São Paulo, Santos 75985-000, Brazil; ronaldo.thomatieli@unifesp.br; 6 Human Movement Laboratory, São Judas University, São Paulo 03166-000, Brazil; 7 São Paulo Futebol Clube, São Paulo 05036-040, Brazil; atitton1@hotmail.com (A.T.); luisflbarros@hotmail.com (L.F.L.d.B.); pedrocuc@terra.com.br (P.C.); felipe.marques@saopaulofc.net (F.O.M.); igorgloria@umc.br (I.P.d.S.G.); 8 Centre of Research and Studies in Soccer (NUPEF), Universidade Federal de Viçosa, Viçosa 36570-900, Brazil; 9 Technological Graduation in Sports and Leisure Management, FATEC of Sports, São Paulo 05818-270, Brazil; vbhirota@gmail.com; 10 Department of Sports Sciences, Polytechnic Institute of Bragança, 5300-253 Bragança, Portugal; jose.eduardo@ipg.pt (J.E.T.); mmonteiro@ipb.pt (A.M.M.); 11 Polytechnic Institute of Guarda, 6300-559 Guarda, Portugal; 12 SPRINT—Sport Physical Activity and Health Research & Inovation Center, 2040-413 Rio Maior, Portugal; 13 Polytechnic Institute of Cávado e Ave, 4800-058 Guimarães, Portugal; 14 Research Center for Active Living and Well-Being (Livewell), Polytechnic Institute of Bragança, 5300-253 Bragança, Portugal; 15 Department of Sports, Higher Institute of Educational Sciences of the Douro, 4560-708 Penafiel, Portugal; 16 Sports Sciences Department, University of Beira Interior, 6200-001 Covilhã, Portugal; 17 Graduate Program in Psychobiology, Universidade Federal de São Paulo, São Paulo 04040-003, Brazil

**Keywords:** soccer, match performance, young players, older players, physical training

## Abstract

**Background:** In elite football, understanding how age impacts players’ physical performance is essential for optimizing training, career longevity, and team management. **Objectives:** This study aimed to compare variations in physical capabilities of professional football players by chronological age and identify peak performance ages. **Methods:** Data from 5203 match performances across 351 official games were analyzed, involving 98 male players aged 18–39 years. Physical capacities (speed, explosive actions, and endurance) were assessed using the Catapult VECTOR7 system. **Results:** showed that players over 32 years experienced declines in high-intensity and explosive actions, while endurance remained relatively stable with age. Peak performance occurred around 25.7 years for speed, 24.8 years for endurance, and 26 years for explosiveness. **Conclusions:** Overall, players aged 17–26 years demonstrated the highest physical performance, with notable declines observed in older age groups.

## 1. Introduction

To understand the influence of age on a professional football player’s career, it is essential to consider various factors that significantly impact performance. Numerous studies demonstrate that age is directly related to performance and longevity [1,2,3]. The natural decline in speed, endurance, and muscular strength with aging affects capacity despite systematic exposure to training and high-intensity matches [4]. Understanding this relationship is crucial in football research, as previous investigations emphasized the importance of skill development, physical fitness, and nervous system characteristics in shaping players’ performance across age groups [5,6]. As players mature, the interaction between physical abilities, technical proficiency, and tactical awareness becomes more pronounced, influencing effectiveness. Thus, training, playing patterns, and age interact to affect outcomes, with more organized football evident at older ages [7,8].

Research on sprint performance and functional abilities in different age groups showed improvements until a peak, emphasizing age’s role in modulating training responses and results [9,10]. Body composition and bone density changes also influence players’ capabilities [11]. The relationship between training load and performance is complex [12,13,14,15], requiring consideration of physical and physiological changes so coaches can adapt programs, optimize capabilities, and mitigate negative impacts [16,17,18].

Moreover, technical and tactical aspects evolve with age, offering insight into holistic development [19,20,21]. Technical proficiency, such as passing, dribbling, and shooting, improves with experience and links closely to physical capacities like speed, agility, and endurance [22]. Evidence also indicates that tactical awareness becomes more refined with age [23,24]. Assessing tactical experience and formation is fundamental in shaping decision-making and strategic contributions. For coaches, detailed knowledge about age effects helps adapt training and tactics to individual needs [25]. For clubs, aging implies issues of squad management and succession planning, requiring a balance between experienced players and youth development [26].

To our knowledge, no study has accurately detailed variations in abilities by chronological age over consecutive seasons. Thus, this study aimed to compare physical capabilities in elite football players stratified by chronological age and identify peak performance ages. We hypothesize that match performance decreases with advancing age due to a blunted response to training load, with younger athletes showing more robust adaptations [27]. We further hypothesize that peak physical performance occurs in the early 20s [28,29].

## 2. Materials and Methods

### 2.1. Subjects

**Figure 1:**
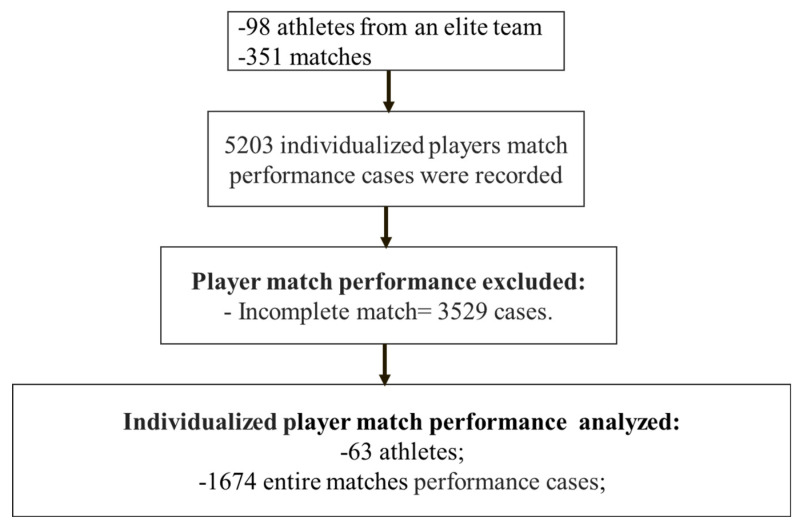
Flowchart with inclusion and exclusion criteria for match performance and training data.

Match performance data (N = 5203 cases) were collected from 99 male professional football players (age 18 to 40 years; weight, 76 ± 6 kg; height, 178 ± 6 cm) belonging to the Brazilian First Division team during the 2020 to 2024 seasons. Data corresponding to 351 official matches from the five seasons were analyzed. The season (with the first official match) starts in January and ends in November or December. Only match performances of the entire match were included in the analysis. This ensures that only starters enter the analysis, thus decreasing bias related to match performance [30] and training load [31]. Figure 1 depicts the inclusion and exclusion criteria for data analysis. After the inclusion and exclusion criteria, the sample was limited to 1674 entire match performances. Players were divided into four positions: striker (159 cases of match performance and respective training load), fullback (572 cases), winger (422 cases), and midfield (521 cases). Goalkeepers were excluded from this analysis due to the different nature of their movement patterns. This study was approved by the São Judas Tadeu University Ethics Committee (Number 6.507.950). This is a retrospective study that assessed data from two seasons of an elite team in the first division of Brazilian football, and it complied with the ethical standards of the University and followed the guidelines of the Declaration of Helsinki.

### 2.2. Data Collection

The catapult system (VECTOR7, Melbourne, Australia) with global and local positioning system devices (GPS, GLONASS & SBAS 18 Hz; LPS, Catapult ClearSky 10 Hz) combined with inertial sensors such as an accelerometer (3D +/− 16G; sampled at 1 kHz, provided at 100 Hz), gyroscope (3D 2000 degrees/second @ 100 Hz), and magnetometer (3D ±4900 µT @100 Hz) were used to collect data for all games. All three inertial sensors collected data on acceleration, force, rotation, and body orientation. The catapult system has suitable validity and reliability for measuring speed, acceleration, and deceleration [32], jump [33], and change of direction [34]. As a previous study [35] identified a small coefficient of variation (0.9 to 1.1%) for intra- and inter-unit reliability, the players used the same device over the season.

The main reason for choosing the variables and their thresholds to conduct this study was to facilitate the understanding of the main idea of this study: to identify the age-related peak performance, stratifying the results by chronological age. Thus, the variables and limits chosen are well studied and well known by coaches in football. Therefore, Inertial Movement Analysis (IMA) method was used to access explosive efforts such as jumps (>40 cm), acceleration (−45 to 0, 0 to 45 degrees), deceleration (135 to 180, −180 to −135 degrees), change of direction (COD) to the left (−135 to −45 degrees) or to the right (45 to 135 degrees). In addition, the total explosive effort (the sum of the jump, acceleration, deceleration, and COD) was recorded as the IMA explosive effort. IMA was used to derive RHIEs (Repeat High-Intensity Efforts: the player performed three explosive efforts in ≤60 s) and RHIE block recovery time (the amount of time to recover and perform another RHIE). The intensity threshold of the IMA event was set when the action occurred at >3 m/s^2^, thus characterizing an explosive action. The running distance producing metabolic power (W·kg^−1^) was also collected at different intensities (>20 and >55 W·kg^−1^). The player load was collected as the sum of the accelerations of the tri-axial accelerometer. GPS methods were used to collect total distance (m), relative distance (m/min), running distance > 20 km/h (m), >25 km/h (m), and >30 km/h. In addition, the number of sprints (running > 25 km/h) and the maximum speed (km/h) achieved during the match were recorded.

### 2.3. Contextual Factors

The players were divided in quartiles (of 5 years for each) for the following reasons: (1) the first quartile (18 to 22 years old) includes players who should be in the club’s youth team but are outliers for their respective ages, thus playing in the main team (professional adult); (2) the second quartile (23 to 27 years old) comprises players that are at the peak of professional performance [28]; the third quartile comprises players with 28 to 31 years old; and the last quartile (>32 years old) considers older players, but they are also outliers for their respective ages, thus still playing in elite football. Therefore, as the players’ ages range from 18 to 39 years old, we divided the players into four groups (five years range each): (1) 18 to 22 years old (27 players and 404 match performance cases); (2) 23 to 27 years old (33 players and 511 match performance cases); (3) 28 to 32 years old (18 players, 541 match performance cases); and (4) >32 years old (11 players; 218 match performance cases). Depending on how players age a year from one season to the next, if the player ages between categories (for example, from the age of 22 to 23), then the 23-year-old’s performances will now belong to the 23- to 27-year-old group. At the same time, the 22-year-old’s performance will be evidenced in the 18- to 22-year-old groups.

The team had six coaches throughout the seasons (coach 1 = 309 cases; coach 2 = 247 cases; coach 3 = 420 cases; coach 4 = 268 cases; coach 5 = 90 cases; and coach 6 = 340 cases) and used the following player positions: fullback, striker, winger, and midfield.

### 2.4. Statistical Analysis

Data is presented as mean and confidence interval (CI) 95%. To examine the impact of age on match performance across five seasons, the performance data of matches were standardized using z-scores within the player position. After standardizing to z-scores, the outliers (less than 1%) were excluded for all performance variables using the ROUT method. Values with a z-score ≥ 3 were excluded. To compare match performance on the age groups, we use the mixed linear model. Also, two contextual factors were used as covariables: player position and coach. Because data from the same player were used multiple times, players were used as random effects (intercept model). To examine the association between player running performance and aging, all match data were standardized to the z-score within each player’s position. First, we performed a hierarchical cluster analysis to generate a dendrogram. Then, based on the visual inspection of the dendrogram created from the hierarchical cluster approach, we identified that three clusters represented the best solution to solve the identified problem (is aging associated with its respective match performance?). Additionally, the best silhouette scores were 0.7 (good) for 2 to 4 clusters, for both speed capabilities and endurance clusters, as well as explosive capabilities clusters. Silhouette scores of 0.5 were achieved for 2 to 4 clusters in change of direction, jump, acceleration, and deceleration capabilities. Thus, it is objectively confirmed that the 3-cluster data set separation is the best solution. Finally, we clustered the match performance based on player position using a k-means approach. The k-means algorithm used Euclidean distance to compute distances and defined 100 iterations to compute the cluster centroids. The reproducibility of the clusters created in this study was tested in the database itself. To complete this, clustering was repeated several times from different classification orders of the database (i.e., changing the initial k centers). As a result, the k-means converged to the same cluster profile regardless of the initial k center, thus demonstrating that our database is sufficiently large and representative of elite soccer players. To validate the clusters of match performance (i.e., to verify whether clusters differed between them), we used one-way ANOVA (with clusters as factors and the z-score of match performance as the dependent variable), followed by the Duncan post hoc test. The η^2^ effect size is reported as ≥0.01 as small, ≥0.06 as medium, and ≥0.14 as a large effect size. All analyses were performed using the IBM SPSS Statistics for Windows (version 27.0, IBM Corp., Armonk, NY, USA). Significance was set at *p* ≤ 0.05.

## 3. Results

### 3.1. Speed Capabilities with Passing Age

**Figure 2:**
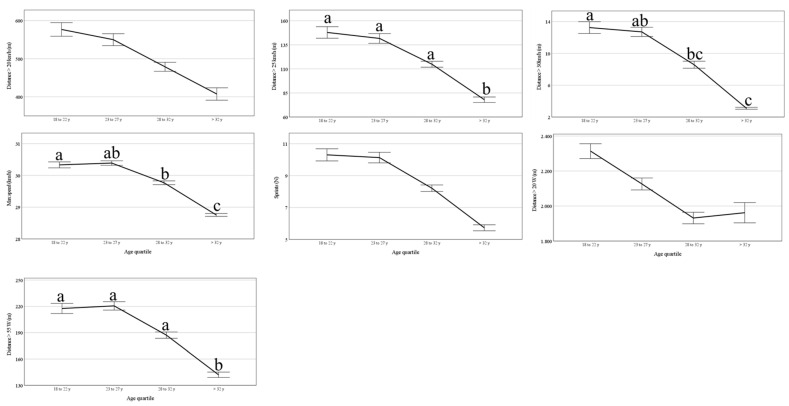
Comparison of the evolution of speed capabilities by age groups. Different letters denote statistical differences between age groups. Data are predicted values and CI 95%.

Figure 2 shows that there is a decrease in performance with advanced age on distance above 25, 30 km/h, 55 watts per kilo of body weight, and maximum speed. On the other hand, there was no difference in less intense speeds, such as distance above 20 km/h, distance above 20 watts per kilo of body weight, and or amount of sprint.

### 3.2. Explosion and Endurance Capabilities with Passing Age

**Figure 3:**
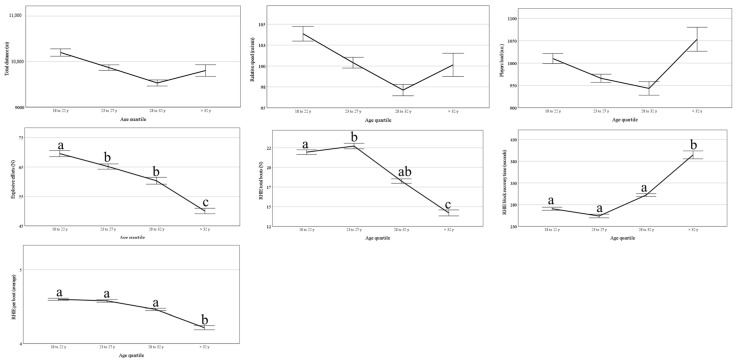
Comparison of the evolution of endurance and explosive capabilities by age group. Different letters denote statistical differences between age groups. Data are predicted values and CI 95%.

Figure 3 shows that, in professional elite football, there is a decrease in explosive football actions with the passing of age, mainly above 32 years of age. On the other hand, there is no change in endurance variables, such as total distance, relative distance, and player load, demonstrating a maintenance of endurance capacity.

### 3.3. Acceleration, Deceleration, COD, and Jump Capabilities with Passing Age

**Figure 4:**
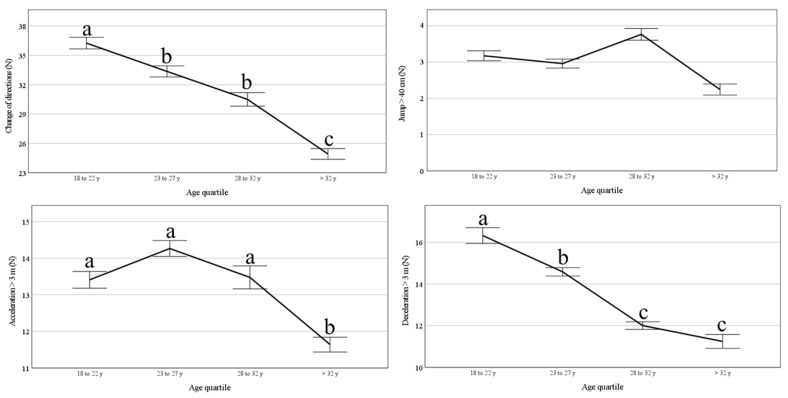
Comparison of the evolution of change of direction, jump, acceleration, and deceleration by age group. Different letters denote statistical differences between age groups in the same season. Data are predicted values and CI 95%.

Our findings indicate that in elite professional football players, with advanced age, there was a significant decrease in acceleration, deceleration, and COD (see Figure 4). However, no chance occurred in the capacity to jump above 40 cm during the football match.

### 3.4. Speed Capabilities Peak Performance According to Age

**Figure 5:**
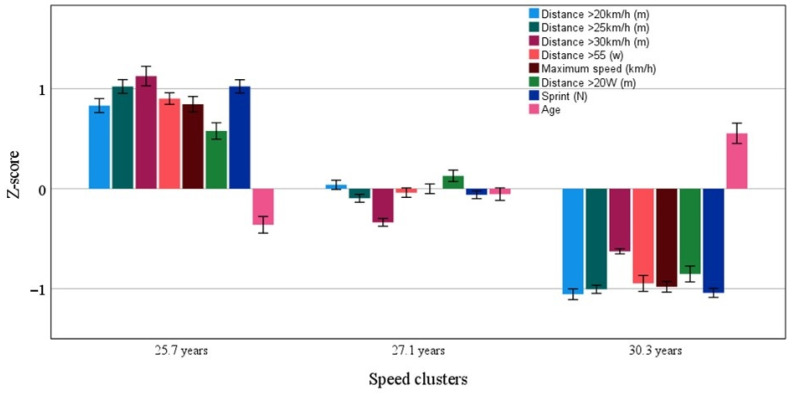
Clusters (mean and 95% CI) of speed capabilities associated with age. All variables are statistically different (all *p* < 0.05) between the three clusters.

With the purpose of identifying the age group with the best performance, the k-means clustering algorithm was employed. In Figure 5, the analysis revealed that clusters at ages 25.7 years old (CI 25.3/26.1 years; Numbers (N) = 415 performance cases) and 27.1 years old (CI 26.8/27.5 years; N = 730 performance cases) have superior performance when compared to the 30.3-year-old (CI 29.7/30.8 years; N = 452 performance cases) cluster. Moreover, the 25.7-year-old cluster had higher performance in all variables when compared to the other two clusters. This methodology allowed us to pinpoint the age group that excelled in speed capabilities.

### 3.5. Endurance and Explosion Capabilities According to Age

**Figure 6:**
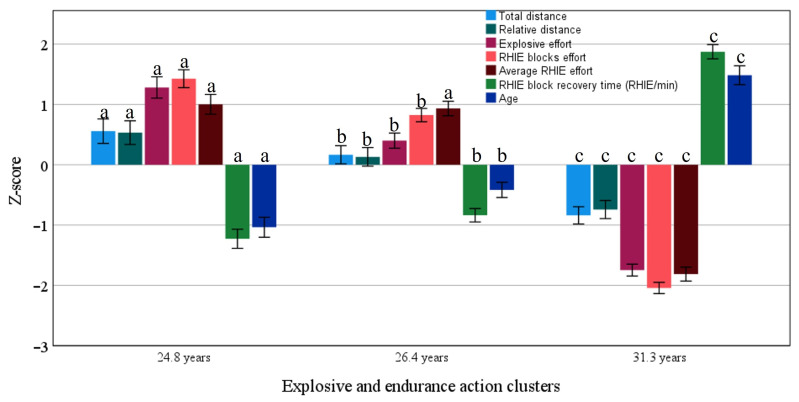
Clusters (mean and 95% CI) of endurance and explosive capabilities associated with age. Different letters denote a statistical difference between the three clusters.

In Figure 6, the k-means identify that the best match performance was from a cluster formed at age 24.8 (CI 24.4–25.3 years; N = 404 performance cases), which presents better performance in the endurance and explosive variables when compared to clusters formed at ages 26.4 (CI 26.0–26.7 years; N = 595 performance cases) and 31.3 (CI 30.9–31.7 years; N = 530 performance cases).

### 3.6. Acceleration, Deceleration, COD, and Jump Capabilities According to Age

**Figure 7:**
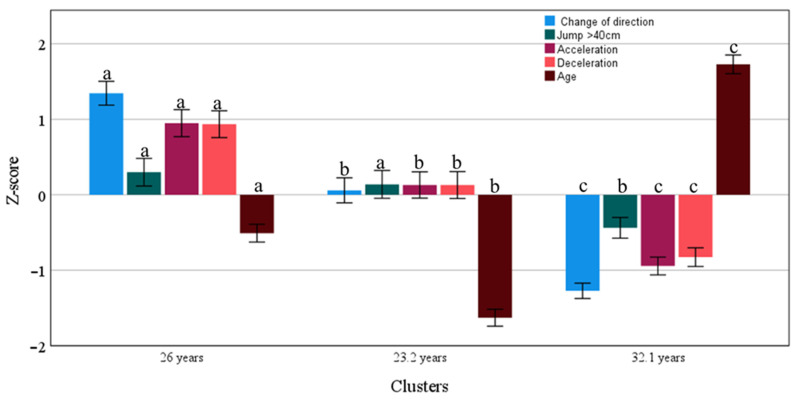
Clusters (mean and 95% CI) of acceleration, deceleration, change of direction, and jump associated with age. Different letters denote statistical difference between clusters.

Figure 7 presents a different level of cluster performance related to age. The cluster related to 26-year-olds (CI 25.7/26.3 years; N = 618 performance cases) has higher performance when compared to 23.2-year-olds (CI 23.0/23.5 years; N = 502 performance cases) and to 32.1-year-olds (CI 31.7/32.3 years; N = 493 performance cases).

## 4. Discussion

The main findings of this study were that match physical performance decreases with advancing age. Additionally, our machine learning analysis suggests that performance peaks in speed, explosiveness, and endurance occur at ~24 to 26 years old.

The division of football players into age groups of 18–22, 23–27, 28–32, and 32+ years aligns well with the reported performance trends observed. Players aged 18–22 have shown consistently higher performance in speed parameters, indicating a phase of rapid development and potential talent acquisition for football clubs [36]. By categorizing players within this age group, clubs can target young prospects who are likely to exhibit growth and improvement over time, making them valuable assets to the team. The >32 y age group represents a stage where performance decline is most pronounced, highlighting the need for clubs to consider alternative roles or support mechanisms for these players [37]. While experience and leadership qualities are valuable assets that older players bring to a team, clubs must also plan for succession and talent development to ensure continuity and competitiveness. Tailored training and workload management for senior players can help prolong their careers and ensure a smooth transition after retirement. A tailored training load seems necessary because no difference in training load was identified across the age categories proposed in this study. (see Table S1).

It is important to note that the technical and tactical performances of these athletes were not assessed. In this sense, future studies should integrate physical, tactical, and technical performance to ensure a more complete view of the effect of age on football performance.

Also, the comparison between the different age groups for both match performance and training load response can provide important insights. In this sense, coaches and trainers should not consider age alone as a determining factor of performance. It is well known that both position and tactical systems significantly impact running performance. In this sense, adjusting the tactical scheme (and changes in the player’s position) to mitigate the effect of age on running performance may be an important strategy to be adopted. Furthermore, as technical and tactical data were not integrated, we cannot rule out the hypothesis that the decrease in external load in match performance identified in older players may be a more efficient response to resolving the demands of the tasks imposed by the game and the coach, and not a decrease in performance resulting from the loss of abilities associated with age. In this sense, future studies integrating technical, tactical, and physical data are necessary to holistically identify the effect of age on football performance.

### 4.1. Speed Capabilities

The analysis of results unveiled a significant age-related influence on speed performance among players. Individuals aged ≥18 to 27 exhibited higher performance across various speed parameters over time, contrasting with those aged >32, who experienced a decline in performance. Leveraging k-means clustering to discern performance cohorts highlighted an optimal age range of around 25.7 years, consistently associated with superior performance across all speed variables. Furthermore, distinct age groups showcased prowess in specific speed facets, underscoring the importance of adaptive training strategies and talent recruitment endeavors that account for age-related variations. These findings furnish valuable insights for athlete development and performance optimization strategies, particularly in sports where speed constitutes a pivotal attribute. Different age groups exhibit prowess in specific speed facets, emphasizing the need for adaptive training strategies and talent recruitment efforts that consider age-related variations in speed performance [37,38,39]. This aligns with the notion that age plays a crucial role in determining the speed capabilities of football players [39,40,41]. Understanding these age-related variations can aid in developing tailored training programs to optimize performance [42,43,44]. The findings from these studies provide valuable insights for athlete development and performance optimization strategies, particularly in sports like football, where speed is a critical attribute [37,45,46,47]. By acknowledging the influence of age on speed performance and implementing targeted training programs, coaches and sports organizations can enhance the overall performance of players and gain a competitive advantage in the field.

In summary, this analysis of the performance of football players across age groups provides valuable insights into the recruitment process of football clubs and the development of age-based coaching strategies. Players aged 25.7 years showed higher performance in several speed parameters over time, indicating potential talent within this age group [36]. On the other hand, players over the age of 32 years experienced a decline in performance, suggesting the need for alternative strategies or a transition to mentoring or coaching roles within the club.

### 4.2. Endurance and Power Capabilities

The results report higher performance in endurance and explosive actions in young age groups. The decrease in performance related to explosive [48] and endurance [48,49] activities is supported by the literature. For instance, the >32 age group experienced longer recovery times between high-intensity blocks, denoting a decrease in capabilities to generate blocks of explosive efforts.

Our primary hypothesis is that a blunted, adaptative response to the training load in the >32-year-old group may have significantly impacted the match performance. The training load was not statistically different between the age groups proposed in this study (see Table S1), a phenomenon that did not occur in the younger groups. Our second hypothesis is that the worsening in explosive actions might be related to overtraining symptoms [50,51], i.e., the inability to adapt to a workload. Therefore, this lack of adaptation in the oldest age group may be related to the inability to respond efficiently to stimuli (mainly from velocity and explosive training). To discard the overtraining symptoms, future studies may solve this issue by quantifying training loads throughout the season (verifying if there are load-dose responses) in the oldest age group.

### 4.3. Acceleration, Deceleration, COD, and Jump Capabilities

The results reveal higher performance of the change of direction (COD), acceleration, and deceleration ability at ~26 years old, while a decrease in performance was registered mainly in the >32-year-old group. This finding is supported by the improvements in specific abilities following training interventions [52].

Jumps were similar between age groups, but the deceleration, acceleration, and COD were evidently higher for the 18–22 age group. This decrease in such parameters aligns with the results of Mcmillan [53], who discussed the challenges faced by football coaches in enhancing aerobic endurance performance during the competitive in-season period. These results emphasized the importance of endurance development in professional football players for maintaining technical and tactical abilities throughout a match [54].

### 4.4. Study Limitations

The present study has some limitations. The data collected from the wearable system may not comprehensively capture all aspects of player performance (for instance, tactical formation). Thus, to mitigate such limitations, we add coaches as a covariable in the mixed linear model. The data from this study come from a team that played eight official competitions with longer travels over the two seasons, and other contextual factors, such as match results, opponent strength, and match venue, which could affect match running performance [54,55] and training load [56], were not controlled. Therefore, future studies should address these issues using a significant data set from a single competition.

Moreover, this study’s findings may not extend to football players from different leagues or countries with diverse playing styles. Variations in training methodologies were not fully accounted for in the analysis, which could influence player performance. The distribution of players across age groups may have influenced the reliability of age-related performance trends. The study’s reliance on data from a single team may limit the generalizability of findings to broader football populations. Future studies could include a longitudinal examination across diverse football leagues and countries to elucidate how performance trends vary based on league dynamics, playing styles, and training methodologies, providing insights into the generalizability of age-related performance trends observed in different football contexts. Additionally, comprehensive analyses of training regimens and interventions employed by professional football teams could be conducted to understand how variations in training intensity, duration, and content influence player performance over time, informing optimal training strategies for enhancing player performance across different age groups. Moreover, exploring the utilization of advanced data collection techniques, such as wearable sensors and machine learning algorithms, to comprehensively capture and analyze various aspects of player performance could offer more detailed insights into the physiological and biomechanical factors influencing player performance and injury risk in football.

Although we use data from the same players over time to identify the effect of aging on athletes and use robust and appropriate statistics, it is important to note that several players left the team due to transfer or injury, and in this sense, a five-season follow-up for the same players is impractical.

## 5. Conclusions

The main objective of this study was to compare the physical capabilities of elite football players, stratifying the results by chronological age. The results indicate that there is a decrease in physical performance during the game with advancing age.

Our main hypothesis is that there is an age-related peak in physical performance in football. Specifically, peak performance might occur in the early 20s [28]. Our results also provide essential support for this hypothesis, and the data indicate that peak running performance occurs at approximately 24.4 to 26.3 years of age. Notably, the age divisions of 18–22, 23–27, 28–32, and 32+ years suggested in this study are appropriate for football clubs’ recruitment processes and the designs of age-based training strategies. These categories align with performance trends observed across different age groups, allowing clubs to optimize player development pathways and make informed decisions to support the long-term success of their athletes. Specifically, the 18- to 22-year-old players, who are outliers for their respective ages, are thus playing on the main team (professional adult) and are improving their physical capabilities. The second quartile (23 to 27 years old) comprises players who are at the peak of their professional performance [28]. The third quartile (28- to 32-year-old players) did not demonstrate superiority in peak running performance; however, it also did not present a fast decline in running performance, such as with the >32-year-old quartile. Finally, the > 32-year-old quartile comprises older players. However, they are also outliers for their respective ages, still playing in elite football, but are far from peak running performance in football.

## 6. Practical Applications

The results of this study provide some practical recommendations that can be made to optimize the management of the physical performance of professional football players throughout the different stages of their careers. Firstly, it is essential that coaches adapt training loads according to the age group of the athletes, especially for players over 32 years of age. For these players, individualized periodization strategies, with greater emphasis on recovery sessions and maintenance of specific skills, can be decisive in prolonging competitive performance.

In addition, the implementation of systematic programs for continuous monitoring of performance and training load is essential. Regular physical assessments, combined with detailed control of training loads, will allow early identification of signs of performance decline or accumulated fatigue, favoring appropriate preventive interventions. In parallel, the results indicate that, from the age of 28, strategies for maintaining speed and explosive capacity should be prioritized, using specific training protocols such as sprints, plyometrics, and agility exercises.

For younger players (18–22 years old), this maturation phase should be used to maximize physical gains, with a focus on developing speed, endurance, and strength, preparing them to reach peak performance. In addition to physical development, the gradual integration of young talent into the first team should be carefully planned, ensuring a continuous renewal of the squad without compromising the experience provided by the more experienced players.

It is also important to note that tactical adaptation should accompany the physical changes resulting from aging. Reductions in acceleration, explosiveness, and speed can be compensated for by adjusting tactical functions and positioning, making the most of the positional intelligence, experience, and game reading of older players. For example, for fullbacks, the coach can ask the defensive midfielder to provide defensive coverage when the fullback moves up to attack. For the midfield, place him as a central midfielder rather than a wide midfielder. This way, the distance to the goal is shorter, and the need for long sprints will be reduced. During the defensive transition, this athlete will only “fit” the marking and will not have such a long return. Another example pertains to the defenders; the coach can mix the defensive duo with an older player (>32 years old) and a young player (~25 years old). Also, when adopting a defensive system with three defenders, it is entirely possible that one of these three defenders is an older player (acting as a central defender). For the attack, if a striker with pivot characteristics is adopted, it is entirely possible for it to be executed by an older player. Finally, given the observed decrease in recovery capacity between blocks of repeated efforts in more experienced players, it is essential to reinforce regeneration strategies, including optimizing sleep, nutrition, and physiotherapy intervention, to preserve the ability to respond to high-intensity efforts.

Based on these recommendations, it is expected that clubs and coaches can more effectively manage their athletes’ careers, favoring the maximization of performance and promoting sporting longevity in high-performance contexts.

Despite some limitations, the study provides valuable insights into the subtle relationship between age and football performance, opening new avenues of research for a more comprehensive understanding of player development and optimization strategies in diverse football contexts. Furthermore, other important contextual variables should be considered in future studies, e.g., (i) game trajectory, (ii) use of data from only one competition, and (iii) qualitative quantification of training load (e.g., whether training consisted of the use of a throw-in, with or without opposition, or a set piece).

## Data Availability

The datasets analyzed for this study can be found at the following link: https://hdl.handle.net/20.500.12682/rdp/GMXME8 (accessed on 2 October 2025).

## Supplementary Materials

The following supporting information can be downloaded at: https://www.mdpi.com/article/10.3390/jfmk10040385/s1, Table S1: Training load comparison between age groups.

## Abbreviations

The following abbreviations are used in this manuscript:

COD	Change of direction
IMA	Inertial Movement Analysis
RHIEs	Repeat High-Intensity Efforts
