# Supplementary material for: The Aging Curve: How Age Affects Physical Performance in Elite Football
Source: J Funct Morphol Kinesiol. 2025 Oct 3;10(4):385. doi: 10.3390/jfmk10040385 (PMC12551122; doi:10.3390/jfmk10040385)
Supplement: Supplementary file 1 [file jfmk-10-00385-s001.zip › jfmk-3872471-supplementary.pdf]

Supplementary Materials

The Aging Curve: How Age Affects Physical Performance in Elite Football

Luís Branquinho [1,2,3,4], Elias de França [5,6,7,*], Adriano Titton [7,8], Luís Fernando Leite de Barros [7], Pedro Campos [7], Felipe O. Marques [7], Igor Phillip dos Santos Glória [7], Erico Chagas Caperuto [6], Vinicius Barroso Hirota [9], José E. Teixeira [10,11,12,13], Pedro Forte [3,4,10,14,15], António M. Monteiro [10,14], Ricardo Ferraz [3,16] and Ronaldo Vagner Thomatieli-Santos [5,17]

1 Biosciences School of Elvas, Polytechnic Institute of Portalegre, 7350-092 Elvas, Portugal; luisbranquinho@ipportalegre.pt
2 Life Quality Research Center (LQRC-CIEQV), 2001-964 Santarem, Portugal
3 Research Center in Sports Sciences, Health Sciences and Human Development, 6200-001 Covilhã, Portugal; pedromiguel.forte@iscedouro.pt (P.F.); ricardompferraz@gmail.com (R.F.)
4 Centro de Investigação do Instituto Superior de Ciência Educativas (CI-ISCE), 4560-547 Penafiel, Portugal
5 Interdisciplinar Graduate Program in Health Sciences, Universidade Federal de São Paulo, Santos 75985-000, Brazil; ronaldo.thomatieli@unifesp.br
6 Human Movement Laboratory, São Judas University, São Paulo 03166-000, Brazil; ericocaperuto@gmail.com
7 São Paulo Futebol Clube, São Paulo 05036-040, Brazil; atitton1@hotmail.com (A.T.); luisflbarros@hotmail.com (L.F.L.d.B.); pedrocuc@terra.com.br (P.C.); felipe.marques@saopaulofc.net (F.O.M.); igorgloria@umc.br (I.P.d.S.G.)
8 Centre of Research and Studies in Soccer (NUPEF), Universidade Federal de Viçosa, Viçosa 36570-900, Brazil
9 Technological Graduation in Sports and Leisure Management, FATEC of Sports, São Paulo 05818-270, Brazil; vbhirota@gmail.com
10 Polytechnic Institute of Bragança, 5300-253 Bragança, Portugal; jose.eduardo@ipg.pt (J.E.T.); mmonteiro@ipb.pt (A.M.M.)
11 Polytechnic Institute of Guarda, 6300-559 Guarda, Portugal
12 SPRINT—Sport Physical Activity and Health Research & Inovation Center, 2040-413 Rio Maior, Portugal
13 Polytechnic Institute of Cávado e Ave, 4800-058 Guimarães, Portugal
14 LiveWell—Research Centre for Active Living and Wellbeing, 5300-253 Bragança, Portugal
15 Sports Department, Higher Institute of Educational Sciences of the Douro, 4560-708 Penafiel, Portugal
16 Sports Sciences Department, University of Beira Interior, 6200-001 Covilhã, Portugal
17 Graduate Program in Psychobiology, Universidade Federal de São Paulo, São Paulo 04040-003, Brazil
* Correspondence: elias.franca@unifesp.br

Data on the conditioning sessions between games were collected. Pre-season training was excluded from the analysis, and a total of 11,658 training sessions were observed. Only the training loads corresponding to complete matches were included.

Table S1: Training load comparison between age groups.

Variables		Age groups	Mean	Standard deviation	95% CI		Difference between age within season
					Lower bound	Upper bound	
Speed variables	Distance >20km/h (m)	18 to 22y	129,9	41,75	107,1	152,7	A
		23 to 27 y	124,5	41,9	101,0	148,0	A
		28 to 32 y	124,3	56,65	93,85	154,7	A
		>32 y	84,6	72,5	30,05	139,1	A
	Sprint (N)	18 to 22y	2,25	1,05	1,65	2,8	A
		23 to 27 y	2,15	1	1,55	2,7	A
		28 to 32 y	1,9	1,45	1,1	2,7	A
		>32 y	1	1,85	-0,35	2,4	A
	Distance >25km/h (m)	18 to 22y	28,7	13,5	21,3	36,1	A
		23 to 27 y	25,8	13,6	18,2	33,4	A
		28 to 32 y	23,9	18,6	13,9	34	A

Variables		Age groups	Mean	Standard deviation	95% CI		Difference between age within season
					Lower bound	Upper bound	
		>32 y	12,4	23,8	-5,5	30,3	A
	Distance >55 (w)	18 to 22y	80,05	23,8	67	93,1	A
		23 to 27 y	78,1	23,8	64,8	91,5	A
		28 to 32 y	70,3	34,2	51,9	88,9	A
		>32 y	57,45	43,6	24,45	90,45	A
	Distance >20W (m)	18 to 22y	654,0	119,8	588,5	719,	A
		23 to 27 y	652,3	120,9	584,4	720,2	A
		28 to 32 y	598,2	153,7	515,5	680,9	A
		>32 y	539,2	198	389,9	688,5	A
	Maximum speed (km/h)	18 to 22y	26,2	1,35	25,45	26,9	A
		23 to 27 y	25,8	1,35	25,1	26,6	A
		28 to 32 y	25,7	1,65	24,8	26,5	A
		>32 y	24,6	2,1	23,05	26,2	A

Variables		Age groups	Mean	Standard deviation	95% CI		Difference between age within season
					Lower bound	Upper bound	
Explosive actions	Distance >30km/h (m)	18 to 22y	2,6	2,5	1,2	3,9	A
		23 to 27 y	1,9	2,5	0,5	3,3	A
		28 to 32 y	1,25	3,6	-0,7	3,2	A
		>32 y	0,2	4,6	-3,3	3,7	A
	RHIE blocks effort (N)	18 to 22y	8,5	2,15	7,4	9,7	A
		23 to 27 y	8,1	2,1	6,9	9,4	A
		28 to 32 y	6,4	2,9	4,8	7,9	A
		>32 y	5,1	3,7	2,4	7,9	A
	RHIE block recovery time (RHIE/min)	18 to 22y	3,8	0,75	3,4	4,2	A
		23 to 27 y	3,9	0,75	3,5	4,3	A
		28 to 32 y	4,05	0,75	3,6	4,4	A
		>32 y	4,8	0,9	4,05	5,5	A
	Average RHIE	18 to 22y	4,2	0,5	3,95	4,4	A

Variables		Age groups	Mean	Standard deviation	95% CI		Difference between age within season
					Lower bound	Upper bound	
		23 to 27 y	4,1	0,4	3,8	4,3	A
		28 to 32 y	3,9	0,5	3,6	4,1	A
		>32 y	3,75	0,65	3,3	4,3	A
	Explosive effort (N)	18 to 22y	33,7	6,5	30,1	37,3	A
		23 to 27 y	32,1	6,5	28,5	35,8	A
		28 to 32 y	26,7	8,15	22,3	31,0	A
		>32 y	26,6	10,5	18,7	34,	A
Endurance actions	Relative distance (m/min)	18 to 22y	62,1	4,9	59,4	64,9	A
		23 to 27 y	62,0	5	59,2	64,9	A
		28 to 32 y	59,5	5,4	56,6	62,4	A
		>32 y	59	7,1	53,6	64,4	A
	Player load	18 to 22y	366,7	43,6	343	390,6	A
		23 to 27 y	364,0	44,0	339,3	388,8	A

Variables		Age groups	Mean	Standard deviation	95% CI		Difference between age within season
					Lower bound	Upper bound	
		28 to 32 y	334,1	52,4	306,0	362,2	A
		>32 y	370,5	67,8	319,5	421,6	A
	Total distance (m)	18 to 22y	3356,35	369,25	3153,95	3558,8	A
		23 to 27 y	3362,9	373,2	3152,4	3573,3	A
		28 to 32 y	17400,4	419,7	2957,0	3410,1	A
		>32 y	3215,3	546,2	2801,3	3629,4	A
Acceleration, deceleration, COD, and jump capabilities	COD (N)	18 to 22y	18,6	4,25	16,3	20,9	A
		223 to 27 y	17,5	4,25	15,15	19,9	A
		28 to 32 y	14,4	5,5	11,4	17,4	A
		>32 y	15	7,1	9,6	20,3	A
	Deceleration (N)	18 to 22y	8,7	1,75	7,8	9,6	A
		23 to 27 y	8,2	1,8	7,2	9,2	A
		28 to 32 y	6,5	1,9	5,4	7,6	B

Variables		Age groups	Mean	Standard deviation	95% CI		Difference between age within season
					Lower bound	Upper bound	
		>32 y	5,9	2,5	3,9	7,8	A
	Jumps > 40cm (N)	18 to 22y	0,7	0,5	0,4	0,9	A
		23 to 27 y	0,6	0,5	0,3	0,9	A
		28 to 32 y	0,8	0,6	0,4	1,1	A
		>32 y	0,4	0,7	-0,1	1	A
	Acceleration (N)	18 to 22y	5,9	1,2	5,2	6,6	A
		23 to 27 y	5,5	1,3	4,8	6,2	A
		28 to 32 y	4,8	1,55	4,05	5,7	A
		>32 y	5,4	2	3,9	6,8	A

The data are the average number of training sessions before the match. Players who did not complete a conditioning session but had played just one match were in the "Difference between age within season" column, and different letters denote statistical differences ($P \leq 0.05$) between age groups. Thus, "A" is different from "B", and the training load represented by "AB" is statistically similar to "A" and "B". #, $P = 0.06$ when compared to the "18 to 22y" age group. In the "age groups" column, the overall effect size ($\eta 2$) is represented by the following colors: small (yellow) and moderate (orange). Overall effect size ($\eta 2$) in column "Age groups" is reported as ≥ 0.01 as small, ≥ 0.06 as medium, and ≥ 0.14 as a large effect size. To compare training load by age, we use a mixed linear model as the fixed effect. Two contextual factors were used as fixed effects: group age and season. Additionally, two contextual factors were used as covariates: player position and coaching staff. Because data from the same player were used multiple times, players were used as random effects (intercept model). All analyses were performed using the IBM SPSS Statistics for Windows (version 27.0, IBM Corp, Armonk, NY, USA). Significance was set at $P \leq 0.05$.
